# It Takes Two–Skilled Recognition of Objects Engages Lateral Areas in Both Hemispheres

**DOI:** 10.1371/journal.pone.0016202

**Published:** 2011-01-24

**Authors:** Merim Bilalić, Andrea Kiesel, Carsten Pohl, Michael Erb, Wolfgang Grodd

**Affiliations:** 1 Department of Neuroradiology, University of Tübingen, Tübingen, Germany; 2 Department of Psychology, University of Würzburg, Würzburg, Germany; 3 Clinic for Psychiatry and Psychotherapy, University of Aachen, Aachen, Germany; Kyushu University, Japan

## Abstract

Our object recognition abilities, a direct product of our experience with objects, are fine-tuned to perfection. Left temporal and lateral areas along the dorsal, action related stream, as well as left infero-temporal areas along the ventral, object related stream are engaged in object recognition. Here we show that expertise modulates the activity of dorsal areas in the recognition of man-made objects with clearly specified functions. Expert chess players were faster than chess novices in identifying chess objects and their functional relations. Experts' advantage was domain-specific as there were no differences between groups in a control task featuring geometrical shapes. The pattern of eye movements supported the notion that experts' extensive knowledge about domain objects and their functions enabled superior recognition even when experts were not directly fixating the objects of interest. Functional magnetic resonance imaging (fMRI) related exclusively the areas along the dorsal stream to chess specific object recognition. Besides the commonly involved left temporal and parietal lateral brain areas, we found that only in experts homologous areas on the right hemisphere were also engaged in chess specific object recognition. Based on these results, we discuss whether skilled object recognition does not only involve a more efficient version of the processes found in non-skilled recognition, but also qualitatively different cognitive processes which engage additional brain areas.

## Introduction

Our object recognition abilities, a direct product of our experience with objects, are fine-tuned to perfection – we need just a split of a second to recognize an everyday object and its function [1]. A dedicated network of left lateralized areas along the ventral and dorsal visual streams has been associated with this amazing feat [2]–[3]. It is less clear, however, whether and how this network enables particularly skilled recognition as found among experts who have extensive experience with domain-specific objects and their functions. Here we show that skilled recognition of chess objects and their functions does not exclusively involve the left lateral areas usually related to normal object recognition. Instead, skilled recognition of chess objects and their functions additionally engage the homologous right regions.

Everyday objects have typical forms that make them recognizable. The ventral visual stream, thought to be essential in object recognition, carries information from the occipital primary visual areas to the inferior-temporal cortex [4]–[5]. A part of the inferior temporal cortex, fusiform gyrus (FG), is thought to mediate the perception of color and form [6]–[7]. The medial part of the left FG participates in everyday object recognition [8]–[9]. Everyday objects, however, have also characteristic functions. This is particularly the case with man-made manipulable objects such as saw or hammer, whose visual features are directly related to their function. These functions are closely coupled to actions which are inevitably associated with movements. The dorsal visual stream is thought to mediate spatially related action [4]–[5]. For example, the posterior middle temporal gyrus (pMTG) at the left lateral side is activated when people name visually or acoustically presented everyday objects, and particularly when they have to retrieve their function [6], [10]–[11]. An explicit retrieval of actions associated with an object is closely associated with the supramarginal gyrus (SMG) in the left inferior parietal lobe (IPL; [3]). The SMG is activated when people are explicitly instructed to retrieve a function-related action with an object [12]–[13] and its activation is particularly modulated by actual execution of an action [14]–[15].

Both, the ventral and the dorsal pathway are more activated in the left than right hemisphere in recognition of manmade objects. This left lateralization probably enables anatomical projections between the object-related brain regions in the left hemisphere [16]–[19].

Although separate characteristics of objects such as form and function engage separate visual streams, recognition of form and function are nevertheless inextricably connected [9]. This reflects our real life experience with objects that are often impossible to recognize without identifying their particular functions. The intrinsic coupling between objects' external features and their functions, as a reflection of our experience with them, has long been recognised in neuropsychology and cognitive neuroscience [10], [20]–[21]. Indeed, a training study of novel objects and their functions [22] indicates that the left lateral (pMTG, SMG/IPL) and left ventral (medial FG) areas mediate recognition of trained artificial objects and their functions.

Although training studies present an excellent way to investigate the impact of prior experience on recognition of objects and their functions, the amount of training in experimental studies is typically limited to a few hours, or in the best case, a few days of training. Yet, in real life, we are exposed to everyday objects for years. From an experimental point of view, it is difficult to measure our experience with objects, and in most cases, we all are familiar with common everyday objects to a similar extent. Here we used the game of chess to circumvent these problems. We employed the expertise approach [23]–[24] to investigate learning related differences in recognition of objects and their functions by comparing expert and novice chess players.

Although chess is a complex cognitive activity that needs years to master [25]–[30], it rests on the recognition of chess specific objects, called pieces, and their functions [31]–[32]. Chess objects are manipulable manmade objects because they have typical forms and shapes that makes them recognizable. The form of a chess object is not directly related to its function but the form and function are firmly coupled through chess rules (e.g., how certain pieces move). The functions are in turn inextricably linked to actions, that is, movements associated with chess objects (e.g., executing a move).

Most importantly, the game of chess enables us to compare chess experts, who possess extensive experience and knowledge about chess objects and their relations, with chess novices, who are superficially familiar with the game of chess and its objects. This expertise approach features falsification in the experimental design [33]–[35] and thus should provide insight in the neural mechanism behind object recognition. Of particular interest is to see how the well-known object recognition network mediates experts' chess specific recognition in comparison to that of novices. Skilled recognition may, for example, engage the same left lateral areas known to be engaged in non-skilled recognition. The same areas would, however, work more or less in terms of increased or decreased neural firing rates to accommodate experience based differences between skilled and non-skilled recognition. In this case, we would assume that skilled recognition involves qualitatively similar processes as non-skilled recognition. The difference would be of a quantitative nature [23], [36]–[37]. Alternatively, the processes of skilled recognition may involve additional areas in the same, or even in the other, hemisphere to meet processing demands. In this case, we would assume that skilled recognition is not only a more efficient version of non-skilled recognition, but that it also involves qualitatively different processes.

**Figure 1:**
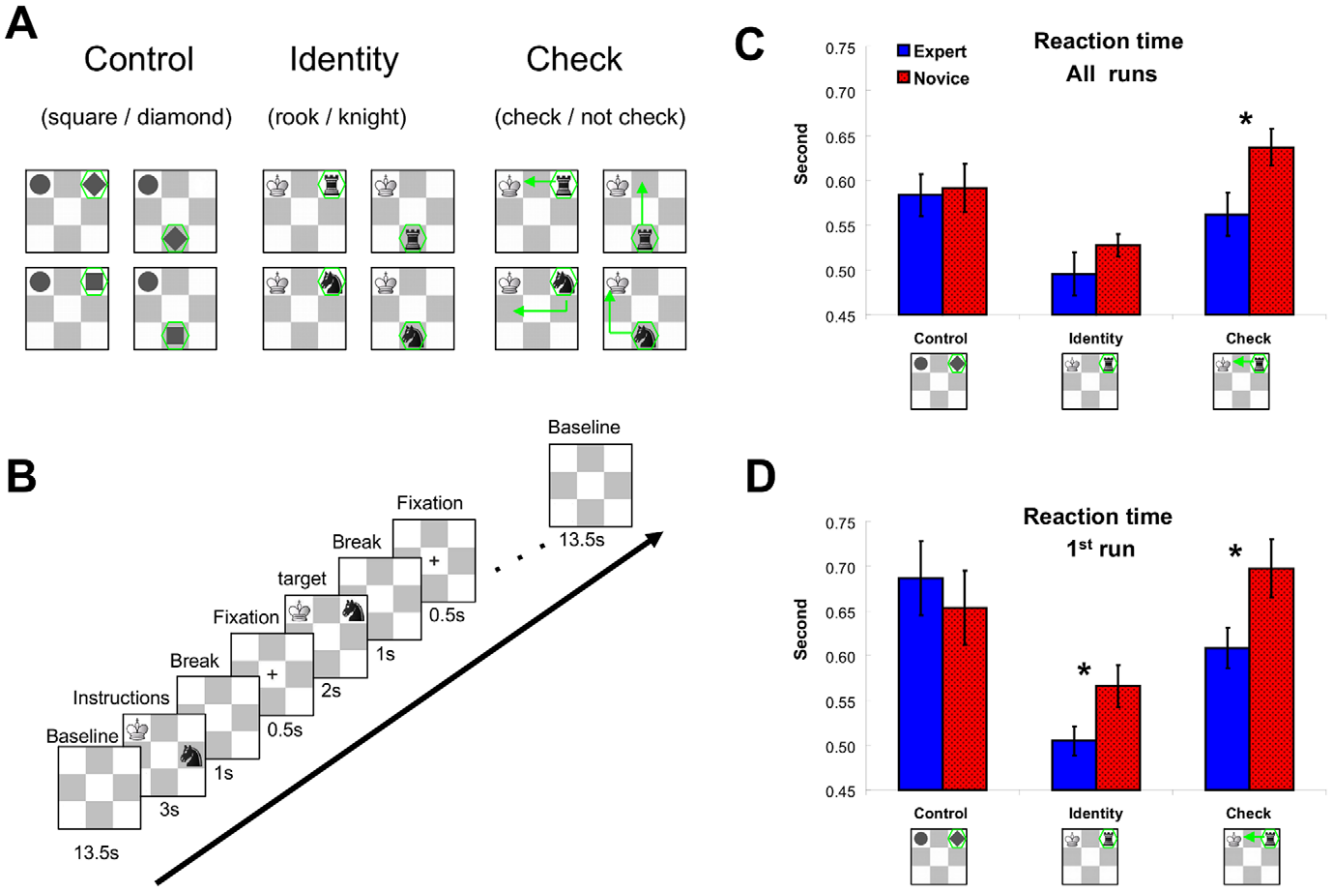
Stimuli and behavioural data. (**A**) Tasks: Control task required to identify geometrical shapes (square or diamond), Identity task to indicate whether the presented piece is a rook or a knight, and Check task to indicate whether the black piece (knight or rook) gives the white king check. The four different stimulus exemplars in each condition are presented (see Methods for explanation). (**B**) Diagram depicting the trial structure. Each block started with an empty 3×3 board, presented for 13.5 s, which acted as a baseline. The baseline was followed by an instruction/task cue for 3 s indicating the required task. After the instruction, an empty 3×3 board appeared for 1 s and served as break. Then a black fixation cross appeared in the middle of the 3×3 board. The cross lasted for 0.5 s and was used to inform players about the upcoming stimulus. The target stimulus lasted until response or maximally for 2 s. Independent on response times, the next trial started 2 s after stimulus onset with the presentation of the 1 s break and the fixation cross. There were 4 trials in a block and after each block the baseline was presented. (**C**) Reaction time (RT; in seconds) averaged for experts and novices in the Control, Identity, and Check tasks over all runs (whole session). (**D**) Reaction time averaged for experts and novices in the Control, Identity, and Check tasks in the first run (first quarter of the session). Error bars indicate the standard error of the mean (SEM). *p<.05 in a two tailed *t*-test for independent samples (experts versus novices).

We investigated the recognition processes of 1) neutral geometrical shapes (Control task), 2) chess objects (Identity task) and 3) functions of chess objects (Check task – see Figure 1A for all three tasks) in expert and novice chess players using behavioral (reaction time and eye movement recordings) and neuroimaging techniques. The Control task involved recognition of geometrical objects because it is reasonable to assume that both expert and novice chess players have the same degree of expertise (most likely rather limited expertise) with geometrical shapes. In contrast, the Identity task required the domain-specific object recognition which should favour experts who possess knowledge about chess pieces (e.g., form, function). Comparing the Identity with Control task will thus enable us to pinpoint behavioral and neural mechanism underlying chess-specific object recognition. The Check task involved object recognition similarly as the Identity task because it is necessary to recognize the chess piece to determine whether it is checking the king. The Check task also required an additional component related to the function of the identified object, that is, the possible moves of this certain piece. The comparison between Check and Control tasks should identify not only object recognition, but also its coupling with the explicit retrieval of objects' functions. The retrieval of function and the process of relating two objects will be identified by comparing Check with Identity task. Finally, the comparison between experts and novices on the chess-specific tasks enabled us to identify the neural basis of skilled chess-specific recognition of objects and their functions.

## Results

### Behavioral Data

Average time in second needed to identify geometrical shapes (Control task), chess objects (Identity task), and check relations of chess pieces (Check task) are shown in Figure 1C for averages over the whole experiment. Given there were learning effects in the task, we were also interested in the performance at the beginning of the experiment. Figure 1D presents the averages over the first run.

#### Chess specific object recognition (Identity vs Control)

The TASK (Control-Identity) × EXPERTISE (Experts-Novices) ANOVA showed that both groups of players needed more time to identify geometrical shapes than chess pieces (main effect task – *F*(1, 14) = 53, *p*<.01). This may be surprising but one must consider the fact that people actually have very limited experience with geometrical shapes. On the other hand, even our novices have probably more often encountered chess pieces than geometrical shapes. The expertise effect (*F*(1, 14) = .8) and the interaction (*F*(1, 14) = .3) were not significant. Although there are descriptive differences between experts and novices in the Identity task, the differences did not reach the significance level (*t*(14) = 1.2, *p* = .26; Control task – *t*(14) = .2, ns.). The reasons for the lack of an expertise effect on the Identity task is most likely a ceiling effect – novices improved their performance over time, while experts were already at the very limits of fast recognition at the beginning of the experiment in the first run (see Figure 1D). To consider untrained task performance we computed the same ANOVA for data of the first run only. The Control task required more time than the Identity task (main effect task – *F*(1, 14) = 28.1, *p*<.01). The expertise effect (*F*(1, 14) = .2) and interaction (*F*(1, 14) = 2.1) were not significant, but single comparisons revealed that experts were significantly faster than novices on the Identity task (*t*-test for independent groups on the Identity task – *t*(14) = 2.2, *p*<.05; Control task – *t*(14) = .6, ns).

#### Chess specific recognition of objects and their functions (Check vs Control)

The time needed to complete the Check and Control task was not significantly different among both groups (main effect of task – *F*(1, 14) = .3), just like any group was not significantly faster over both tasks (main effect of expertise – *F*(1, 14) = 3.1). Experts were, however, much faster than novices on the Check task, while there were no differences on the Control task (task x expertise interaction – *F*(1, 14) = 29.6, *p*<.01; *t*-test for independent groups on the Check task – *t*(14) = 2.4, *p*<.05). The same pattern of results was obtained when the first run was separately analyzed (task x expertise interaction – *F*(1, 14) = 10.7, *p*<.01; *t*-test for independent groups on the Check task – *t*(14) = 2.5, *p*<.05; main effect task – *F*(1, 14) = .2, ns; main effect expertise – *F*(1, 14) = .6, ns).

#### Chess specific recognition of functions (Check vs Identity)

Finally, the Check task was more demanding than the Identity task as indicated by increased RTs for the Check task (main effect task – *F*(1, 14) = 91.7, *p*<.01). The difference between experts and novices was more pronounced on the Check task than on the Identity (task x expertise interaction – *F*(1, 14) = 7.5, *p*<.05). The main effect of expertise did not reach significance (*F*(1, 14) = 3, *p* = .10), because of the small differences in the Identity task whereas experts' and novices' performance differed in the Check task. As mentioned above, we attribute the lack of the significant expertise effect to a ceiling effect. In the first run, that is without training on the tasks, the reaction times were increased in the Check task compared to the Identity task (main effect task – *F*(1, 14) = 49.5, *p*<.01), and experts were faster overall (main effect expertise – *F*(1, 14) = 6, *p*<. 05). There was no significant interaction (*F*(1, 14) = .3, ns.).

The superiority of experts on the chess specific tasks is not a product of their disregard for accuracy. If anything, experts made fewer mistakes in the chess related tasks (see File S1).

### Eye Movement Data

Experts were faster in domain specific recognition of objects and their functions, but the advantage is not driven by their superior general recognition (there were no differences in the Control task). This pattern of results points to a highly efficient and domain specific mechanism underlying experts' superior recognition of objects and their relations. Eye movements will further elaborate and shed light on the nature of the mechanism [23], [38]–[39]. We were particularly interested in the pattern of fixations (i.e. percentages of fixation on objects of interest). The number of fixations followed the behavioural analysis (see File S1).

**Figure 2:**
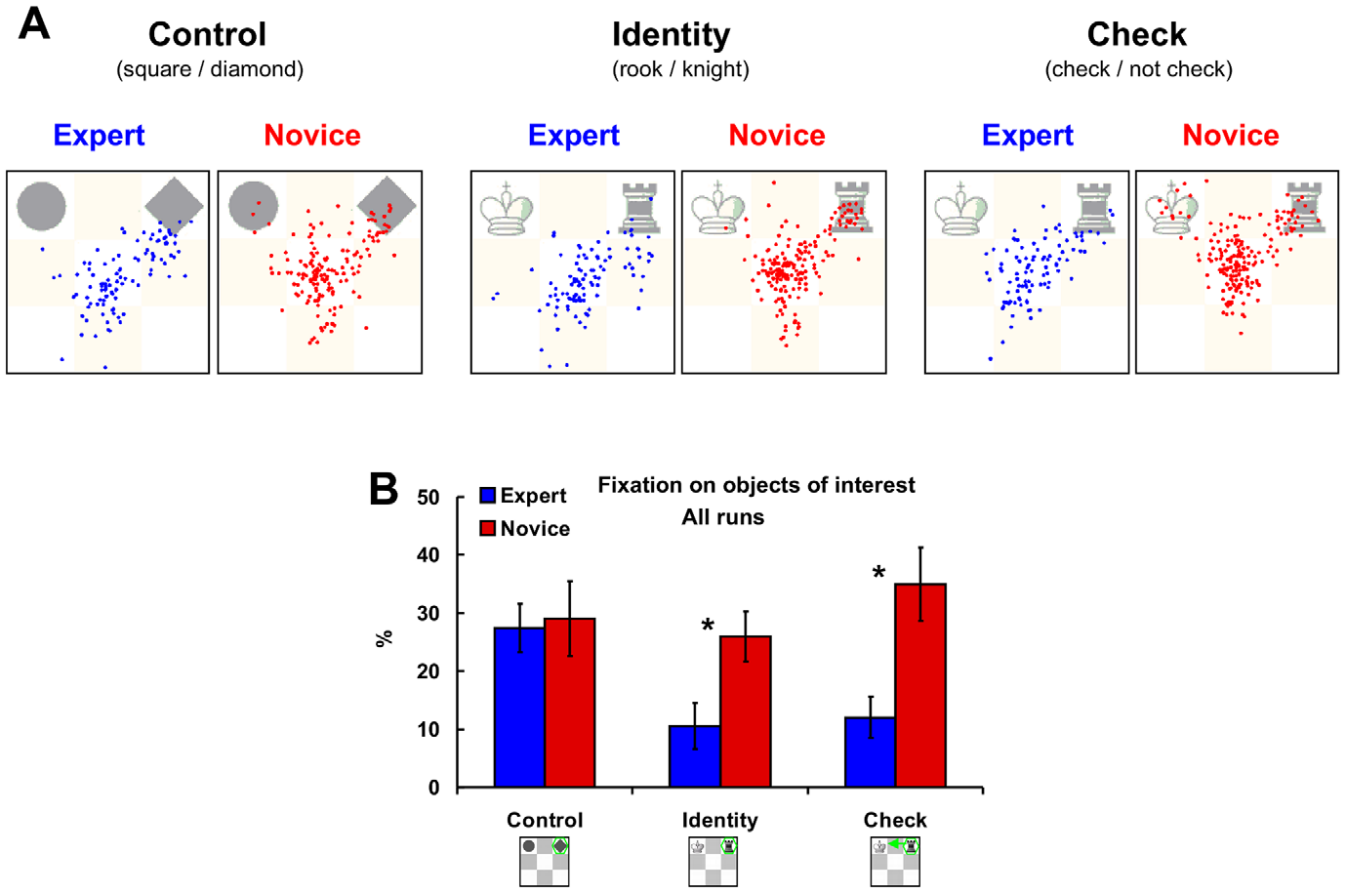
Eye movement data. (**A**) All fixations of experts (blue dots) and novices (red dots) on an example stimulus in the Control (left), Identity (middle), and Check (right) task. (**B**) Average percentage of fixation that falls on objects of interests in a trail in experts and novices in the Control, Identity, and Check tasks averaged across all runs. Error bars indicate SEM. * *p* <.05 and †*p* <.10 in a two tailed *t*-test for independent samples (experts versus novices).

Figure 2A shows that experts and novices did not only differ regarding the number of fixations for the three tasks, but also on the pattern of fixations. When the stimuli were not chess specific in the Control task, the eye movements of experts and novices were similar (Figure 2A, left panel). In contrast, in the Identity task novices fixated more often directly at the chess piece they needed to recognize, while experts fixated beside the pieces and at the centre of the board (Figure 2A, middle panel). The differences were also evident in the Check task (Figure 2A, right panel) as novices needed to attend to both pieces to make sure that the function of the chess objects forms the check relation. The pattern of fixations in experts remained the same as in the Identity task – they fixated mostly at the centre of the board.

#### Chess specific object recognition (Identity vs Control)

We calculated the percentage of fixations that fell on the squares with objects of interest (pieces needed to complete the tasks) to statistically confirm these observations. Figure 2B shows that overall in all runs players tended to fixate more often on the objects of interest in the Control task than in the Identity task (main effect task – *F*(1, 9) = 4.6, *p* = .06). This was mostly related to the performance of experts on the Identity task as they fixated considerably less often directly on the objects of interests. Although neither the main effect of expertise (*F*(1, 9) = 3.4) nor its interaction with tasks (*F*(1, 9) = 2.7) were statistically significant, experts fixated less often directly on objects of interest than novices on the Identity task (*t*(9) = 2.4, *p*<.05; Control task – *t*(9) = .5, ns.).

#### Chess specific recognition of objects and their relations (Check vs Control)

Similar results were obtained when we compared Check and Control tasks. Fewer direct fixations were found on the Check task, but mainly because of the performance of experts on the Check task. The main effects of expertise (*F*(1, 9) = 3.2, *p* = .11) and task (*F*(1, 9) = .6, ns.) were not significant just like their interaction (*F*(1, 9) = 3.1, *p* = .11). Experts fixated less often directly than novices at the objects in the Check task (*t*(9) = 2.6, *p*<.05).

#### Chess specific recognition of functions (Check vs Identity)

In both chess tasks novices fixated objects of interest more often directly than experts (main effect expertise – *F*(1, 9) = 7.4, *p*<.05). The main effect of task (*F*(1, 9) = 1.3) and interaction (*F*(1, 9) = .2) were not significant.

### Neuroimaging Data

Eye movement analysis showed that expert players do not directly focus at the chess objects to identify them unlike novices who directly fixated on the chess objects to perform the chess tasks. The neuroimaging data will provide the neural mechanism behind experts' superior recognition of objects and their functions.

#### Chess specific object recognition (Identity vs Control)

**Figure 3:**
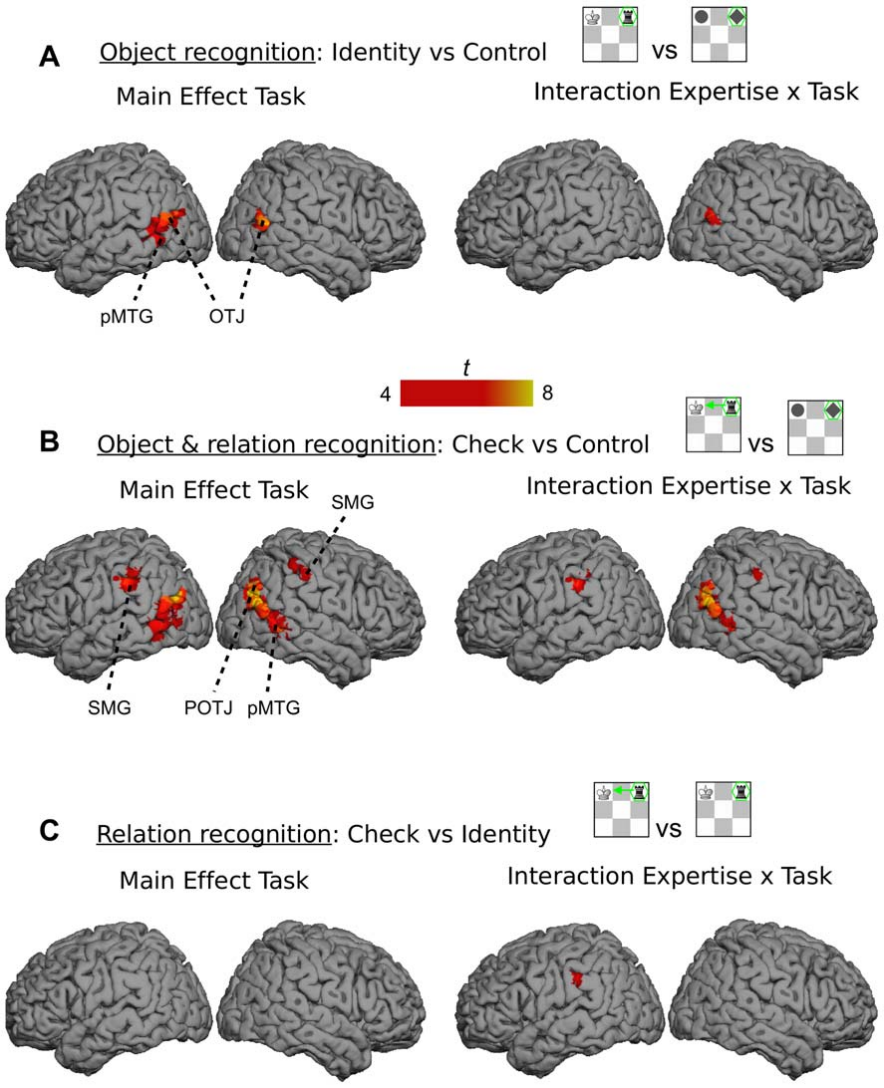
Neuroimagining data. (**A**) The network of brain areas activated in recognition of chess object across all runs (whole session) – contrast Identity vs Control task (left side) and its interaction with expertise (right side). (**B**) The network of brain areas activated in recognition of chess object and their functions across all runs (whole session) – contrast Check vs Control task (left side) and its interaction with expertise (right side). (**C**) The network of brain areas activated in recognition of object functions across all runs (whole session) – contrast Check vs Identity task (left side) and its interaction with expertise (right side). The comparisons were based on *p*<.05 (corrected) and clusters of 5 or more voxels. The interaction between task and expertise in (A) and (C) were based on a lower threshold of *p*<.00001 (uncorrected). The significant areas included bilateral posterior middle temporal gyrus (pMTG), bilateral occipito-temporal junction (OTJ), right parieto-occipito-temporal junction (POTJ), and bilateral supramarginal gyrus (SMG). The MNI coordinates can be found below the labels of the ROIs in Figure 4.

The direct comparison of Identity and Control task will provide information regarding the neural basis of chess specific object recognition (main effect of task). We were also interested in the areas that showed different sensitivity for experts and novices in the Identity but not in the Control task (task x expertise interaction). Figure 3A shows that the left lateral areas such as pMTG and the neighbouring occipito-temporal junction (OTJ) were significantly more activated in the Identity than in the Control task. Besides these left lateral areas, the right OTJ junction was also more activated in the Identity task. There were no significant areas for the tasks x expertise interaction at the corrected threshold. When we slightly lowered the threshold to *p*<.00001 (uncorrected), we found the right OTJ activated (right side of Figure 3A). All other areas, including the inferior temporal lobe associated with recognition of color and shape, revealed no significant activation related to chess specific object recognition. There were also no significant effects of expertise. This is not surprising because the comparison (main effect of expertise) involves a neutral control task for which there should be no differences.

#### Chess specific recognition of objects and their functions (Check vs Control)

We further compared the Check and Control tasks to identify brain areas associated with not only recognition of objects, but also recognition of relations between them. The comparison between Check and Control tasks (main effect task) revealed significant activations in the areas as in the previous comparison – left lateral areas (pMTG & OTJ) and right OTJ (Figure 3B). Additional brain areas were also activated – supramarginal gyrus (SMG) at the inferior parietal lobe (IPL) at the left hemisphere, and pMTG, parieto-occipito-temporal junction (POTJ), and SMG at the right hemisphere. The task x expertise interaction revealed activation only in the right lateral areas and the left SMG (right side of Figure 3B). Again, experts and novices did not reveal differently activated brain regions across both tasks, which is not surprising given that the differences were not expected in the Control task.

#### Chess specific recognition of functions (Check vs Identity)

To elaborate which areas are exclusively related to the recognition of relations between objects we compared the Check and Identity tasks. There were no areas that differentiated between the two tasks (Figure 3C) and no areas were sensitive to the interaction between tasks and expertise. When we lowered the threshold to *p*<.00001 (uncorrected), the left SMG was active (right side of Figure 3C). In both tasks experts engaged more the right OTJ than novices (main effect of expertise).

**Figure 4:**
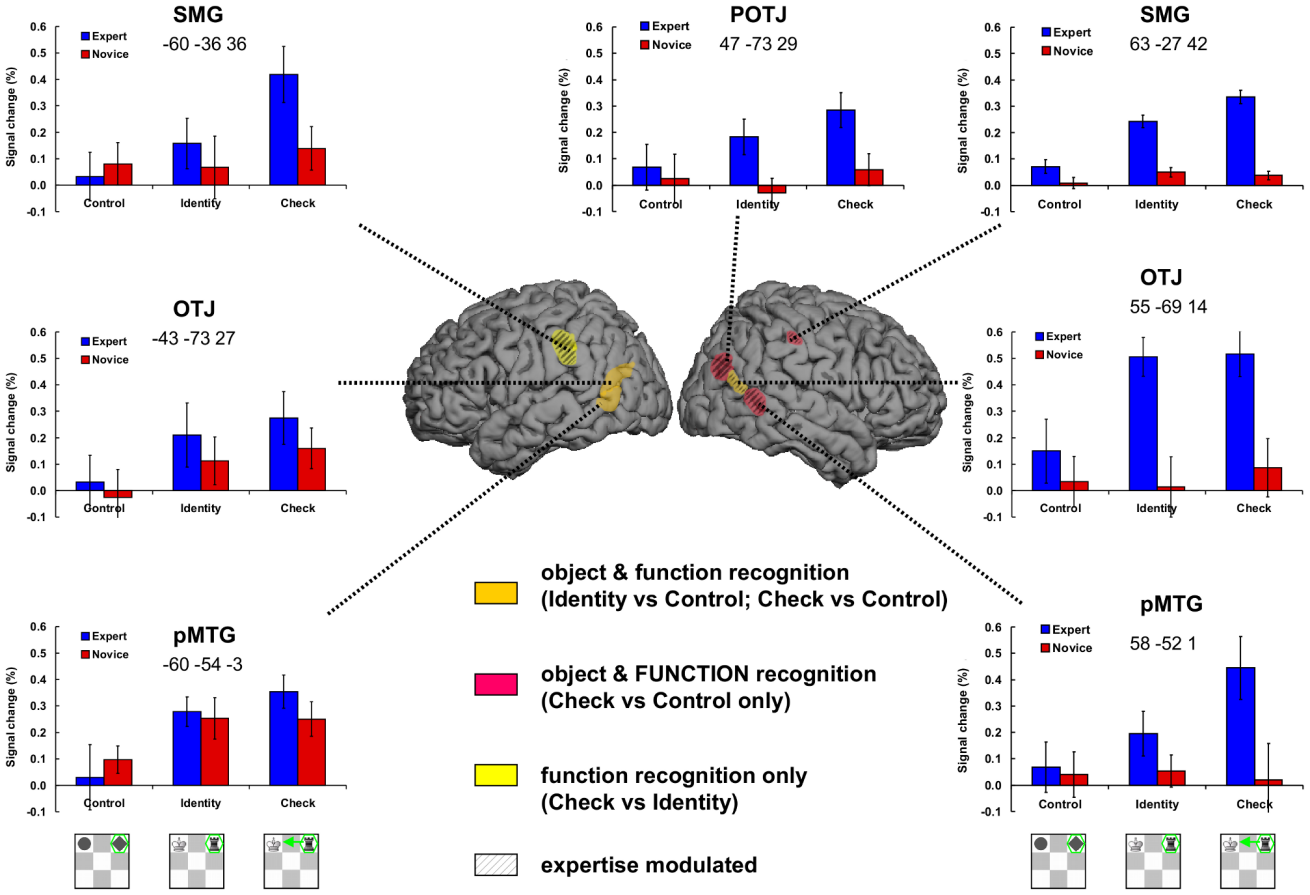
Neuroimagining data summary. Presents the object specific network based on the three comparisons in Figure 3. Orange color indicates the areas activated in both Identity vs Control, and Check vs Control task comparisons (both separately, not conjunction analsysis); Red indicates the areas activated only in the Check vs Control task (FUNCTION is in capital letters to emphasize that this area explicitly involves function of objects, unlike the areas in orange); Yellow indicates the region significantly activated in the Check vs Identity. Please note the colours are transparent and on the surface of the brain image may look slightly different. The areas modulated by expertise have additional black stripes. In each of these areas the regions of interests (ROI) were taken by selecting the voxels within 3 mm^3^ of the peak activation (see MNI coordinates below the ROI labels). The activation levels (percent signal change relative to baseline) were extracted for each individual player and averaged across groups and tasks for the first run only (similar results were obtained when all runs were used).

Comparing the fMRI data of players on the chess tasks (Check and Identity) and the Control task we found a network of brain areas responsible for the recognition of chess objects and their functions. Figure 4 summarizes the findings and plots activation levels in the first run in these areas. While this confirms the whole brain analysis, it additionally provides an overview of the results [40]. Just like the recognition of other manipulable man-made everyday objects with clearly specified functions [3], recognition of chess objects and their functions was left lateralized. The left tempo-lateral areas were more activated in the Check and Identity tasks than in the Control task. Chess recognition, however, also engaged additional right lateral areas, but only in experts. The activation of novices on both chess-specific tasks in the right lateral brain areas was similar to their activation on the Control task. The only other area that was affected by expertise was the left SMG, which was more engaged among experts than among novices in the Check task. The left SMG was also the only area that was significantly more responsive in the Check than Identity task, presumably indicating its relevance in retrieval of object functions and establishing relations between objects [2]–[3].

### Relation Between Behavioral, Eye Movement and Neuroimagining Measures

In this study, we were interested in the differences between experts and novices and designed a task that captured these differences on behavioral, eye movement, and neural levels. We consider all three levels as indicators of the same underpinning mechanism responsible for superior recognition of objects and their relations among experts (for a discussion and justification of this view, see [36], [41]). However, it is justified to question whether the differences at the neural level are a pure reflection of expertise the way they are at the behavioral and eye movement level (see [36] for a review). More specifically, neuroimagining results could be confounded by the differences in reaction time, eye movements, and task difficulty. We believe such confounds are unlikely for the following reasons. First, as mentioned in the method section, controlling for reaction time did not produce a different pattern of results in the fMRI analysis. Second, the number of eye movements per se cannot account for neural differences because an additional, second control task that required more eye movements than any other task, did not significantly differ in the level of activation from the control task presented here (see File S1). Third, the second control task was also more difficult than the chess tasks as indicated by reaction time (see File S1). And yet, activation levels in the expertise modulated areas were smaller than in the chess specific tasks.

## Discussion

We demonstrated the influence of experience related knowledge on the recognition of objects and their relations at behavioral and neural levels. Expert chess players were faster on the chess-related tasks than novice players. The eye movement analysis showed that experts' greater knowledge about chess specific objects and their functions enabled them to recognize chess objects and relations between them with fewer fixations and without directly fixating on the objects. In contrast, novices required more fixations in general with a higher proportion of direct fixations. Novices were consequently slower in recognizing chess objects and their functions. Experts' advantage was chess specific as it disappeared on the control task featuring neutral geometric shapes. The neuroimaging data related the chess specific object recognition to the bilateral areas of the dorsal stream in the lateral temporal and parietal lobes.

Our results underline the importance of experience and knowledge on object recognition. Novices were competent players and had no difficulties in distinguishing between different chess pieces as indicated by a small number of errors (see File S1) and rather fast reaction times (Figure 1C–D). And yet, novices were clearly slower than experts at arguably basic chess object recognition tasks. The difference was particularly pronounced in the Check task when players, in addition to identifying the chess object itself, had to retrieve its function and relate it to another chess object. The behavioral results are in line with previous behavioral chess studies [31]–[32], [42].

### Neural Basis of Skilled Object Recognition

The neuroimaging data provide additional confirmation of functional importance in object recognition. Chess specific object recognition was associated exclusively with the action related dorsal visual stream. Both temporal and parietal lateral areas of the dorsal stream were associated with chess specific recognition of objects and their relations (see Figure 4). As previously mentioned, the left temporo-lateral areas have been found to be associated with object recognition in numerous studies (for reviews, see [2]–[3], [43]). Further, evidence that left temporo-lateral areas play an essential role is provided by patient studies. The impairments in these areas are accompanied with the inability to recognize and/or manipulate objects [44]–[45]. Our results show that chess specific object recognition is also tied to the same areas, thus providing a further generalization to a new kind of man-made objects. The bilateral parietal areas (SMG) were also related to object recognition, in particular to retrieval of object function and recognition of relations between two objects as required in the Check task (see Figure 4). These parietal areas are important in action related processing as evident by their activation when participants retrieve and/or execute actions [3], [12]–[15], or when participants passively observe the action of man-made objects [46].

The activation in both temporal and parietal lateral areas was more pronounced in the Check task, which required action-related retrieval of function, than in the Identity task, which required only object recognition (see Figure 4). None of these regions, however, were significantly more activated when the Check task was directly compared with the Identity task (see Figure 3C, left panel). These results should be considered carefully in the light of this study. On the one hand, the absence of brain areas that distinguish between the Check and Identity tasks may be taken as further evidence that the recognition of object is inextricably and automatically coupled with their functions. On the other hand, it is possible that our experimental design did not have enough power to detect such, presumably small, effects. Finally, the left SMG showed a significant modulation by expertise indicating that parietal regions may be more specialized for functional properties of objects in experts.

### Right Hemisphere Involvement in Skilled Object Recognition

The left temporo-lateral areas were engaged by both experts and novices to a similar extent. The homologous right brain temporo-lateral areas were, however, also activated in chess specific object recognition. Moreover, these right areas distinguished between experts and novices, unlike their homologous left counterparts. The right temporo-lateral areas also did not seem to be of much use for novices as evidenced by the similar activation levels on the chess specific and the control tasks.

Differences in spatial attention can hardly account for the expertise effects. Although effects of spatial attention are generally right lateralized, they engage different areas in the temporal and parietal lobe [47], [48]. The right temporo-parietal junction, associated with switching of attentional focus [47] is located superior to the expertise related temporal areas and inferior to the SMG. Similarly, other regions thought to be involved in spatial attention, such as superior parietal lobe (SPL), intraparietal sulcus (IPS), and precuneus [47], were not related to expertise effects – even in a less stringent whole brain analysis.

It is also difficult to directly relate the differences in the patterns of fixation to the expertise-related activation differences in right lateral brain areas. The stimuli were centrally presented and the distance between the starting fixation at the center of the board and the objects of interest was less than 2°. If a kind of parafoveal or peripheral vision was at play, the differences between experts and novices were not observed in brain areas that are commonly related to peripheral vision, such as medial areas of the inferior temporal lobe [49]. A less stringent whole brain analysis did not reveal expertise-related activation differences in these medial areas of the inferior temporal lobe.

Most likely the patterns of fixations and the engagement of the right lateral brain areas in expert chess specific recognition are related to the same underlying cause – the chess specific knowledge. Experts have developed knowledge structures through extensive exposure to chess stimuli [28], [50]–[55]. These sophisticated knowledge structures not only involve information about types and location of chess objects [28], [50]–[51], but also about the relations between these objects through objects' function [55]. When presented with two or more chess objects, knowledge structures enable experts to automatically and in parallel retrieve functions of chess objects and thus rapidly examine if the objects are in (check) relations [39], [56]–[58]. Novices do not possess extensively developed knowledge structures and, although they are familiar with chess objects and their functions, the retrieval of functions and relations between pieces probably takes place in a serial nature and thus considerably slower [31]–[32], [39], [56]–[58].

The question remains why the right temporo-lateral brain areas are associated with skilled object recognition. One possible explanation would be the holistic processing of stimuli which is generally more related to the right hemisphere than to the left one [59]–[60]. Right hemisphere, for example, processes more global aspects of a visual stimulus, while left hemisphere is better in processing local aspects [61]–[63]. The skilled recognition of chess objects and their function, however, also involved the left hemisphere. Although there were some differences between the activation levels in right and left hemispheres among experts, the activations in both hemispheres were clearly above the baseline (see Figure 4). It is thus possible that in experts both hemispheres may work together to enable automatic and parallel processing and thus superior domain specific object recognition. The engagement of both hemispheres to meet additional task demands is well researched in attention and working memory [64]–[65]. Simple tasks may require only single hemisphere regions, but more demanding complex tasks additionally engage homologous areas in the other hemisphere [66]. Similarly, experiments on visual laterality show that lateralized processing is sufficient in simple tasks, but more complex tasks are solved better when both hemispheres contribute [67]–[68]. In these instances the cost related to the communication between hemispheres is offset by the benefits of the processing in both hemispheres [66].

The exact mechanisms of the inter-hemispheric communication are less clear. They could involve, for example, parallel independent processing as well as highly dependent processing through inter-hemispheric interaction [66], [69]–[70]. These mechanisms could increase the processing power by allocating different computations involved in the task to different hemispheres.

The inter-hemispheric interaction offers a plausible explanation for the bilateral activations in chess experts. Although the tasks in our study were relatively simple and seemingly required little effort, the eye movements showed that experts used a different processing strategy than novices. Experts' strategy is arguably more difficult and only possible because of the extensive chess specific knowledge. The well-known parallel and automatic processing among experts [39], [56] may require additional brain resources for successful execution. In the case of skilled chess specific object recognition these additional computational resources are located in the homologous right temporal and parietal areas. The nature of our design makes it difficult to connect each hemisphere and their specific temporal and parietal areas to specific processes that are required for the chess specific recognition (e.g., recognizing an object, its function, and relating it to a different object). A promising approach could be a more direct manipulation of involved brain regions by excitation or inhibition through transcranial magnetic stimulation (TMS).

The finding that experts' object processing is supported by additional homologous brain structures, however, may indicate that there are qualitative differences in skilled and non-skilled chess object recognition. The involvement of both hemispheres is also found in other paradigms in the same domain [23], [71]. It is also in line with observations in other domains that skilled processing qualitatively differs from novices' processing. Experts in mental calculations, for example, also additionally engage homologous brain areas in comparison to novice mental calculators when presented with a demanding task [72]. Thus, experts, supported by their knowledge base, are able to employ more sophisticated and efficient processing strategies when necessary. This reasoning corresponds with numerous behavioral and eye movement studies that demonstrate the qualitative differences between experts' and novices' cognitive processing [39], [73]. The present study suggests that the qualitative nature of different processing strategies is also reflected in different patterns of brain activations.

### Conclusion

Our expertise approach combined with concurrent application of behavioral and neuroimaging techniques enabled us to uncover cognitive and neural mechanisms underlying skilled object recognition. We showed that skilled recognition is not solely based on more efficient versions of the same cognitive processes necessary in non-skilled recognition. Instead, skilled recognition may involve qualitatively different cognitive processes which are accommodated in the human brain through engagement of additional homologous brain areas. This finding is important because it may reflect a general characteristic of expertise. It also underlines the importance of investigating the cognitive processes in experts because many of them may not reflect only a more efficient version of the processes we normally find in lay-people.

## Materials and Methods

### Participants

Eight expert chess players (mean age ± standard deviation, 29±7 years) and eight novice chess players (29±5) participated in the experiment. The size of the expert sample corresponds to the expert samples used in behavioral research on expertise [31], [38], [74]–[76] and is larger than the few neuroimagining studies involving chess experts [71], [77]–[78]. Most importantly, our experts were exceptionally skilled practitioners. Players get rated based on their performance against other rated players. The international chess Elo scale is an interval scale with a theoretical mean of 1500 and standard deviation of 200 [79]. Experts are players with a rating of 2000 Elo points or more. Our experts were highly rated – on average 2130 (+/−147) points – and were thus highly skilled chess players. Novice players were hobby players who played chess occasionally. Their chess skills were clearly inferior to experts but they had no difficulties in identifying chess pieces and their functions (these aspects are the absolute basics of the chess game). The novices would easily beat beginners who usually struggle to relate chess objects to their typical function. All players were male and right-handed. The Institutional Review Board of the Ethic Committee of Tübingen University approved this study and written informed consent was obtained from all participants.

### Tasks and Stimuli

Each player performed three tasks. In the first Control task players had to indicate if the stimulus presented was a diamond or a square by pressing the left or right button, respectively (see File S1). In the Identity task, players had to identify if the presented black chess piece was a rook or a knight (by pressing the left or right button, respectively). In the Check task, players were provided with the same stimuli as in the Identity task, but now they had to indicate if the black chess piece was giving check to the white king (left for check and right for no check). Stimuli were an artificial 3×3 chess board with two chess pieces. In the Control task, a grey circle was always presented in the upper left corner that was irrelevant for the task and was presented to keep the visual complexity of the stimuli similar to the Check task. An additional object, a grey square or diamond, was presented at the upper right location or the lower middle location (see Figure 1A). In the Identity task, the geometrical shapes were replaced by a black knight or rook while the circle was replaced by a white king. The white king had no function in this task and was presented to ensure visual compatibility between this and the Check task. The knight and rook appeared at the same two locations as the geometrical shapes – upper right and lower middle location. The Check task involved the same stimuli as the Identity task (king always at the upper left location and knight or rook variably at upper right or lower middle location). The king now had to be taken into account to solve the task successfully.

There was an additional Control task (Control 2) that we present in File S1. This Control task had the same geometrical shapes for the stimuli as the other Control task, but required taking into account two features, location and shape, for successful execution. Players had to indicate whether there was a circle on a grey location or a square on a white location (one response category) or whether there was a circle on a white location or a square on a grey location (the alternative response category). The task was used to additionally control for attention and eye movements (see File S1).

The dimension of the whole stimulus was 150×150 pixels, while the dimension of a single square was 50×50 pixels. The stimuli were projected onto a screen above the head of the players via a video projector in the adjacent room. Players saw the stimuli through a mirror mounted on the head coil (see File S1). The physical dimensions of the stimulus were 126 mm for the whole stimulus and 42 mm for the single square. The setup resulted in a visual field of 7.2° for the whole stimulus and 2.4° for a single square on the board.

### Design and Procedure

The tasks were performed in blocks consisting of four trials with the same task. In a single run there were 16 blocks, four for each task (including the additional control task – see File S1). There were four runs. The order of blocks within a run was randomized and counterbalanced over players. Each block started with an empty 3×3 board that acted as a baseline (see Figure 1B). The baseline of 13.5 s was followed by an instruction screen of 3 s indicating the task. After the instruction, there was a break for 1 sec in which an empty 3×3 board was presented. Then a black cross appeared in the 3×3 board. Players were asked to fixate the cross. The cross lasted for 0.5 s and was used to inform players about the upcoming stimulus. The trial stimulus was then presented for maximum of 2 s. Players were allowed to move their eyes during the trial. The trial stimulus immediately disappeared after the response was given and was replaced by a break which filled the reminding two seconds (e.g., if the response was given after 0.5 s, the break lasted for 1.5 s). After the trial stimulus (and the break), the 1 second-break and the fixation cross were again presented (to enable a break between the trials and as a warning for the next stimulus, respectively). Each block contained 4 trials and blocks were separated by the baseline and the instruction. Response times were measured from stimulus onset until onset of the key press. In all tasks, players indicated their decision by pressing one of two buttons of an MRI-compatible response device held in the right hand (the left button was for diamond, rook, or check, depending on the task, and the right button for square, knight, no check). All players first read the instruction and were presented in advance with a practice block for each task outside of the scanner. Before the actual session, players practiced each task for two blocks.

### Eye-Movement Data Acquisition

Players' eye movements were recorded by an infra-red remote long-range eye-tracking device (iView X MEyeTrack Long Range, SMI) sampling at 50 Hz. The device is MRI-compatible and did not interfere with players' performance (see File S1). The system had an error of 0.5–1°, corresponding to 8.6–17.1 mm (that is less than a third of a square) on the board. We used a nine-point calibration with bi-quadratic functions before each run. We created a program in MatLab 7.1 to analyze the eye movement data of four experts and seven novices (technical problems prevented eye movement measurement in the other players). We defined a fixation as an event where players kept their eyes within a diameter of 25 pixels for 80 ms or more. Using a larger diameter to define a fixation did not markedly influence the results presented here and in File S1. We extracted the fixations for each player on each stimulus in each task. These fixations were then averaged across stimuli, tasks, and groups (see File S1). In order to investigate the pattern of fixations, we calculated the percentage of fixation that fell within a square where an object of interest was placed. In the Control and Identity tasks, the square of interest was always the square where the geometrical shape or chess piece was located; while in the Check task it was always two squares – that of the chess object and the constant square of the king (upper right).

### Behavioral and Eye Movement Analysis

Since we were interested in differences between the individual tasks, we compared performance in the Identity task and the Control task using a 2 (task – Control/Identity) ×2 (expertise – Experts-Novices) ANOVA. Two 2×2 ANOVAs were computed to compare Check – Control tasks, and Check – Identity tasks. Because we were mainly interested in the difference between experts and novices in all the tasks separately, we further computed *t*-tests for independent samples, separately for all three tasks.

### Imaging Data Acquisition

We acquired fMRI data using a 3 T scanner (Siemens Trio) with a 12-channel head coil at the fMRI center in Tübingen, Germany. We covered the whole brain using a standard echo-planar-imaging sequence with the following parameters: [TR] = 2.5 s; [FOV] = 192×192; [ET] = 35 ms; matrix size  = 64×64, 36 slices with thickness of 3.2 mm+0.8 mm gap resulting in voxels with the resolution of 3×3×4 mm^3^. Anatomical images covering whole brain with 176 sagittal slices were obtained after the functional runs using an MP-RAGE sequence with a voxel resolution of 1×1×1 mm^3^ (TR = 2.3 s, TI = 1.1 s, TE = 2.92 ms).

### Functional MRI Data Analysis

The preprocessing was done with SPM5 and involved spatial realignment to the mean image including unwarping, co-registration of the anatomical image to the mean EPI, unified segmentation procedure, normalization to the MNI-brain template and a 8-mm FWHM spatial smoothing. We modelled the blocks for each tasks in each run together with the instruction explicitly while the baseline was implicitly modelled in a general linear model (hemodynamic activation modelling relied upon a canonical response function, AR(1) and a 128 Hz high-pass filter). The fMRI analyses equalled the analysis of behavioral and eye movement data. First, we compared Identity with Control task filling the parameters (con images) of the individual analysis of each player in a 2×2 ANOVA. The results of the main effects and their interaction were examined at a significance level at *p*<.05 (FWE; corrected for multiple comparisons; 5 or more contiguous voxels). Further 2×2 ANOVAs were computed to compare Check and Control tasks, and Check and Identity tasks. The results are presented in Figure 3 using Surfrend Toolbox in SPM5 and FreeSurfer. Including the average RT of each player as covariate at the second level, as a way of controlling for the influence of reaction time on BOLD signal, produced the similar pattern of results as presented in Figure 3.

For illustrative and descriptive purposes, we used the MarsBaR SPM Toolbox (Marseille ROI toolbox, Version .041) to extract the signal percent change relative to baseline in each task in the first run for each participant (see [40]). Since the significant regions were close to each other, we used a 3 mm^3^ sphere around the most activated voxel as regions of interest (ROIs) for each of the significant areas (see Figure 3 in the main text). The descriptive results from the first run (all runs produce a similar pattern of results) are presented in Figure 4 together with a summary of the fMRI analyses. Using all runs did not change the pattern of activation levels.

## Supporting Information

File S1 **Additional data, analysis, and seven figures.**
(DOC)
