# Supplementary material for: It Takes Two–Skilled Recognition of Objects Engages Lateral Areas in Both Hemispheres
Source: PLoS One. 2011 Jan 24;6(1):e16202. doi: 10.1371/journal.pone.0016202 (PMC3025982; doi:10.1371/journal.pone.0016202)
Supplement: File S1 — Additional data, analysis, and seven figures. (DOC) [file pone.0016202.s001.doc]

**Supplementary Information**

It Takes Two – Skilled Recognition of Objects Engages Lateral Areas in Both Hemispheres

Merim Bilalić Andrea Kiesel Carsten Pohl Michael Erb Wolfgang Grodd

**Supporting Method**

**Tasks, stimuli and apparatus.** The Control task involves artificially manmade objects (geometrical shapes), but in this setting they do not have a particular function. In contrast, the Identity and Check task involve artificially manmade objects (chess pieces) which have clearly specified functions. Comparing chess-specific tasks with the Control task enables us to pinpoint the neural basis of general chess-specific recognition of objects and their functions among both experts and novices. The experimental setup is presented in Fig. S1.

There was an additional Control task (Control 2) that used the same geometrical shapes as stimuli as the other Control task, but required taking into account two features, location and form, for successful execution. Players had to indicate whether there was a diamond on a grey location or a square on a white location (one response category) or whether there was a diamond on a white location or a square on a grey location (the alternative response category). The task was used to additionally control for effort and eye movements.


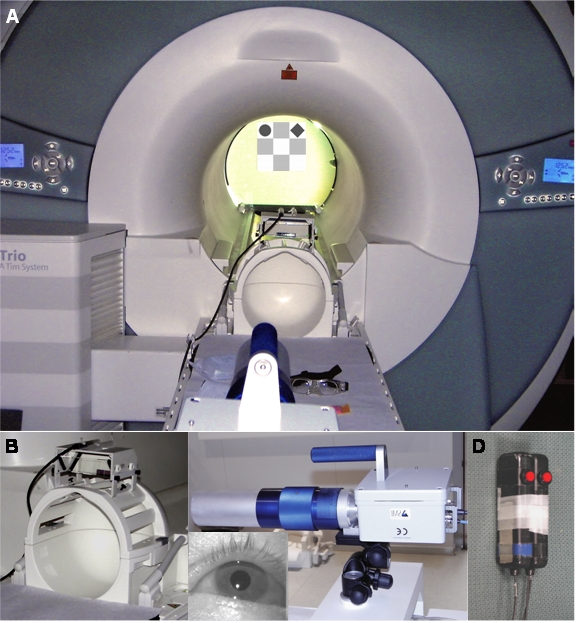


***Fig. S1*. Experimental Setup.** (a) Front view of the Siemens Trio 3T scanner used in the experiment with a chess position projected. (b) Twelve-channel head coil with the mirrors and the light source for the eye tracker. (c) The iView X MEyeTrack Long Range (LR) infra-red remote long-range eye-tracking camera. (d) Response buttons used in the study.

**Supporting text and analyses**

**Behavioral results.** We used the second Control task (Control 2) to additionally control for difficulty and eye movements (see method in the main text for the description of the task). Fig. S2 shows that participants responded slower in Control 2 task than in the other three tasks regardless whether we analyze all runs or just the first one. The differences between Control 2 on the one side, and the other three tasks on the other, were significant (*t*(15) = 11, *p* < .001; *t*(15) = 11.2, *p* < .001; *t*(15) = 8.1, *p* < .001; for Control 2 versus Control, Identity, and Check, respectively across all runs, and *t*(15) = 5.9, *p* < .001; *t*(15) = 9.8, p < .001; *t*(15) = 6.1, *p* < .001*.*; for Control 2 versus Control, Identity, and Check, respectively in the first run). There were no differences between experts’ and novices’ RTs on the second control task (*t*(14) = .2, *ns*). Differences in experts and novices regarding the other tasks are described in the main text.


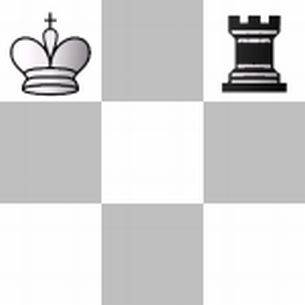

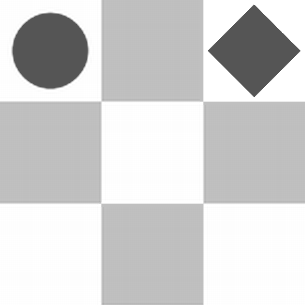

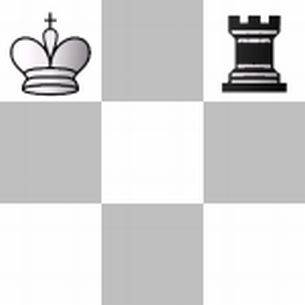

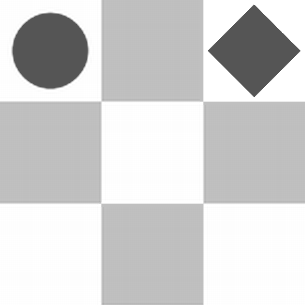


**Reaction Time**

**All runs**

**Reaction Time**

**1^st^ run**


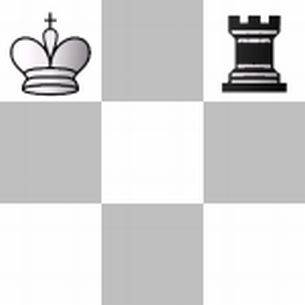

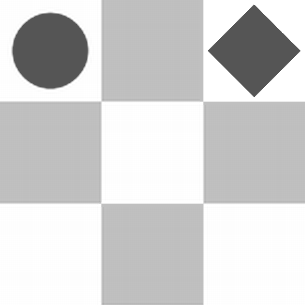

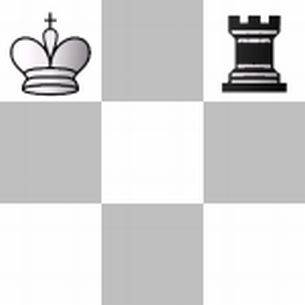

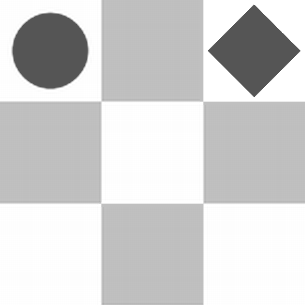


*****

*****

*****

***Fig.S2*.** Time needed to complete the tasks across all runs (left) and in the first run (right). The right side of the bold line presents the three tasks used in the main text. The results are the same as in the main text and were used here to compare them with the second control task. Error bars indicate the standard error of the mean (SEM). **p* <. 05 in a two tailed *t*-test for independent samples (experts versus novices).

The players made less than 10% errors in all four tasks (see Fig. S3). Experts tended to make less errors than novices in the Check task (right panel), but the difference, as well as the difference in the other tasks, did not reach the significance level (across all runs and in the first run only).

Similar to RTs, the error rates indicate that the second control task was the most difficult task. Players made significantly more errors in Control 2 than in the other tasks, with the exception of the Check task in the first run where there was no significant difference (*t*(15) = 3.6, *p* = .003; *t*(15) = 6.4, *p* < .001; *t*(15) = 4.1, *p* = .001; for Control 2 versus Control, Identity, and Check, respectively across all runs, and *t*(15) = 2.6, *p* = .021; *t*(15) = 4.1, *p* = .001; *t*(15) = 1.2, *ns*; for Control 2 versus Control, Identity, and Check, respectively in the first run).

***Fig. S3*.** Percentage of errors made across all runs (left) and in the first run (right). The right side of the bold line presents the three tasks used in the main text. Error bars indicate SEM.

**Eye movement results.** Fig. S4 shows the fixation of experts (blue) and novices (red) on a stimulus from the second control condition.

***Fig. S4*.** All fixations of experts (blue dots) and novices (red dots) on an example stimulus in the Control 2, Control, Identity, and Check task.

The eye movement analysis of number of fixations confirms that the second Control task was more difficult than the other three tasks (Fig. S5). Players made more fixations in Control 2 task than in the other three tasks (*t*(10) = 3.9, *p* = .003; *t*(10) = 5.1, *p* < .001; *t*(10) = 3.8, *p* = .001; for Control 2 versus Control, Identity, and Check, respectively across all runs, and *t*(10) = 3.8, *p* = .003; *t*(10) = 4.9, *p* = .001; t(10) = 3.4, *p* = .006; for Control 2 versus Control, Identity, and Check, respectively in the first run). There were no differences among experts and novices on the second Control task. All other differences are reported in the main text.


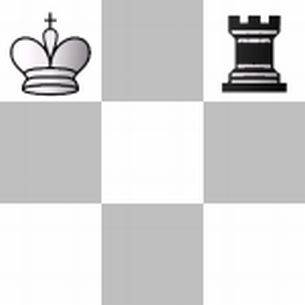

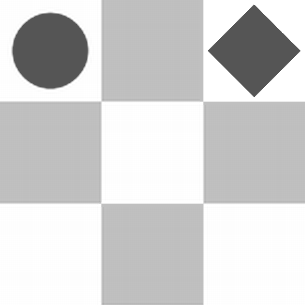

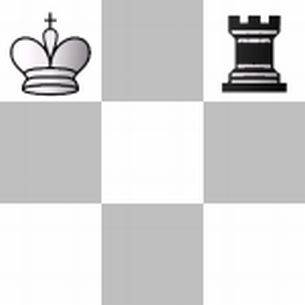

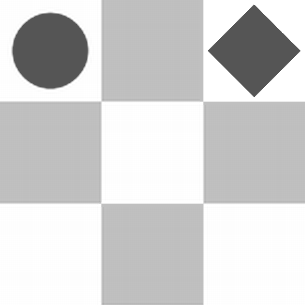

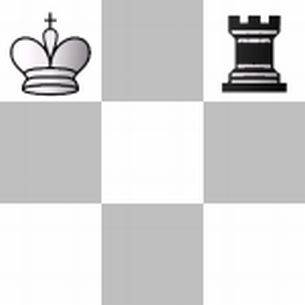

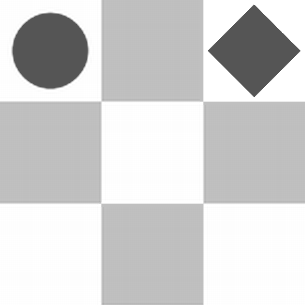

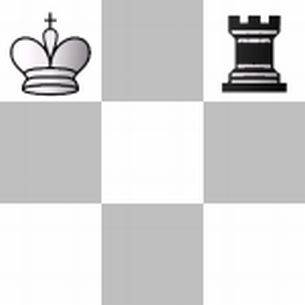

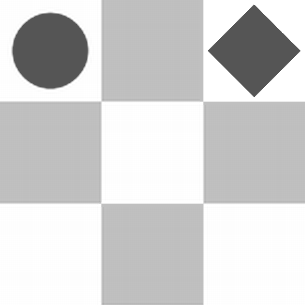


**All runs**

**Fixation**

**1st run**

**Fixation**

*****

*****

*****

**†**

***Fig. S5*.** Number of fixations needed to complete the tasks across all runs (left) and in the first run (right). The right side of the bold line presents the three tasks used in the main text. The results are the same as in the main text and were used here to compare it with the second Control task. Error bars indicate SEM. **p* <. 05 and †*p* <. 10 in a two tailed *t*-test for independent samples (experts versus novices).

When we analysed the percentage of fixation falling on the squares of interest, we found the players fixated more often directly at the objects of interest in the second control task (Control 2) than in the other task but some of the differences did not quite reach the significance level (*t*(10) = 1.8, *p* = .11; *t*(10) = 3.8, *p* < .005; *t*(10) = 2.2, *p* < .05; for Control 2 versus Control, Identity, and Check, respectively across all runs, and *t*(10) = 1.2, *p* = .25; *t*(10) = 2.2, *p* < .05; t(10) = 1, *p* = .34; for Control 2 versus Control, Identity, and Check, respectively in the first run). There were no differences among experts and novices on the second Control task in the first run only and across all runs. For other differences see the main text.

***Fig. S6*.** Percentage of fixations on the objects of interest across all runs (left) and in the first run (right). The right side of the bold line presents the three tasks used in the main text. The results are the same as in the main text and were used here to compare them with the second Control task. Error bars indicate SEM. **p* <. 05 and †*p* <. 10 in two tailed *t*-tests for independent samples (experts versus novices).

**Neuroimaging results.** The main effect of expertise in the three comparisons in the main text (Fig. 3) did not produce any significant areas at the set level (*p* < .00001). This is not surprising because the contrast involve the comparison on the Control task, for which no differences between experts and novices were expected. The interaction between task and expertise disentangles the influences of different tasks and is presented in the main text (see Fig. 3).

The second Control task was more difficult than the original Control task. It required more effort and attention as evidenced by the data on reaction time and errors (see Fig. S2-3). Players also needed more eye movements in the second Control task than in the original Control task (see Figure S4-5). If the identified brain regions were sensitive to attentional effects (reaction time, errors) and eye movements, one may expect different level of activation in the second Control task in comparison with the original Control task. Fig. S7 shows that this is not the case – there were no differences in the activation levels between the two Control tasks in the identified brain regions when the first run was taken into account (no difference were obtained across all runs either). The effects in the identified brain regions thus do not reflect attention and/or eye movements but rather other chess-specific differences.

***Fig. S7***. Activation levels (relative to baseline) in the first run. The right side of the bold line presents the three tasks used in the main text. The results are the same as in the main text and were used here to compare them with the second Control task.
